# The Multifaceted Zoonotic Risk of H9N2 Avian Influenza

**DOI:** 10.3390/vetsci5040082

**Published:** 2018-09-21

**Authors:** Elizabeth A. Pusch, David L. Suarez

**Affiliations:** Southeast Poultry Research Laboratory, US National Poultry Research Center, Agricultural Research Service, US Department of Agriculture, 934 College Station Road, Athens, GA 30605, USA; Liz.Pusch@ARS.USDA.GOV

**Keywords:** H9N2, avian influenza, zoonotic, human infection

## Abstract

Poultry-adapted H9N2 avian influenza viruses (AIVs) are commonly found in many countries in Asia, the Middle East, Africa, and Europe, and although classified as low pathogenic viruses, they are an economically important disease. Besides the importance of the disease in the poultry industry, some H9N2 AIVs are also known to be zoonotic. The disease in humans appears to cause primarily a mild upper respiratory disease, and doesn’t cause or only rarely causes the severe pneumonia often seen with other zoonotic AIVs like H5N1 or H7N9. Serologic studies in humans, particularly in occupationally exposed workers, show a large number of people with antibodies to H9N2, suggesting infection is commonly occurring. Of the four defined H9N2 poultry lineages, only two lineages, the G1 and the Y280 lineages, are associated with human infections. Almost all of the viruses from humans have a leucine at position 226 (H3 numbering) of the hemagglutinin associated with a higher affinity of binding with α2,6 sialic acid, the host cell receptor most commonly found on glycoproteins in the human upper respiratory tract. For unknown reasons there has also been a shift in recent years of poultry viruses in the G1 and Y280 lineages to also having leucine instead of glutamine, the amino acid found in most avian viruses, at position 226. The G1 and Y280 poultry lineages because of their known ability to infect humans, the high prevalence of the virus in poultry in endemic countries, the lack of antibody in most humans, and the shift of poultry viruses to more human-like receptor binding makes these viruses a human pandemic threat. Increased efforts for control of the virus, including through effective vaccine use in poultry, is warranted for both poultry and public health goals.

## 1. Introduction

Low pathogenicity avian influenza virus (LPAIV) subtype H9N2 is the most prevalent LPAIV in poultry in the world [1,2,3]. Although wild waterfowl are the natural host of avian influenza, H9N2 is a relatively uncommon subtype in wild birds. The first H9N2 virus in domestic poultry was isolated from turkeys in the 1960s in the United States of America (USA) and only sporadic reports from poultry were reported until the 1990s [4]. In the mid-1990s H9N2 was first reported from chickens in Guangdong Province of China, and subsequently was detected throughout the country [5]. An unrelated H9N2 outbreak was reported from South Korea in 1996 that also became endemic [6]. In the late 1990s, the detection of H9N2 was reported from a number of different countries in Southeast Asia and the Middle East, and now H9N2 has become enzootic in many Asian, Middle Eastern, and North African countries [7,8]. The poultry-adapted H9N2 viruses have become a major concern for poultry health in the last 20 years, but has also become a concern for human health as some of the H9N2 lineage viruses are zoonotic [1,9,10,11,12].

All H9N2 viruses are considered low pathogenic avian influenza viruses based on the lack of mortality in the standardized in vivo pathotyping test in specific pathogen-free (SPF) chickens [13]. H9N2 infections in poultry in the field are quite different in that birds show mild-to-severe respiratory disease signs, decreases in egg production, and in some cases up to 20% mortality [14]. The difference in the more severe clinical disease observed in the field compared to the laboratory is thought to be caused by co-infection with other pathogens including mycoplasma and infectious bronchitis virus, immunosuppressive infections with viruses like infectious bursal disease virus, and stressful environmental conditions including high temperature or high ammonia levels.

## 2. H9N2 Genetic Lineages

While the H9 hemagglutinin subtype is associated primarily with the N2 neuraminidase subtype in poultry, it can also be naturally found with other neuraminidase subtypes. The H9N2 avian influenza subtype lineages that are now endemic in poultry likely have several different origins. Currently there is not a consensus in the scientific community on how the different lineages should be referred to and this clearly adds to the confusion when discussing different isolates. For the purposes of this review, the viruses circulating primarily in Korea, first isolated in 1996, will be referred to as the Korean lineage [6]. Some researchers refer to this group as the Y439 lineage based on a duck isolate from Hong Kong isolated in 1997, but this virus is at best only distantly related to the Korean isolates and is not an appropriate reference strain. The second major poultry lineage found primarily in China includes 3 different reference strains, A/Chicken/Beijing/1/1994, A/Chicken/Shanghai/F/1998, and probably the most common reference being to A/Duck/Hong Kong/Y280/1997. This lineage is extremely diverse and a clear source of origin is not apparent, and will be referred to as the Y280 lineage to follow most published reports [15,16]. The third major group, and also the most divergent group, is the G1 lineage with the reference strain of A/Quail/Hong Kong/G1/1997. Although the reference virus appears to be one of the first isolates within this lineage of virus, it is clear when making phylogenetic comparisons that multiple sublineages were already established in several different countries by as early as 1998, and the G1 Hong Kong virus is likely not the progenitor for this entire lineage of viruses. However, for simplicity the G1 nomenclature will be used for this group of viruses that have become endemic in India, Pakistan, the Middle East, and North Africa and recently into two countries in sub-Saharan Africa. A fourth poultry adapted lineage has been circulating in Europe since at least 2013 primarily in the turkey industry [17,18]. Other wild-bird H9 lineage viruses have also been reported.

## 3. Evolution of H9N2

Like all avian influenza viruses (AIVs), H9N2 is prone to genetic changes that can affect virulence, pathogenicity, and host specificity. Antigenic drift caused by the lack of a proof-reading mechanism for influenza A viral RNA polymerase leads to high genetic variability from point mutations in the nucleotide sequence [19]. These point mutations can lead to changes in the amino acids of the viral genes, of particular interest are those on the surface of the hemagglutinin and neuraminidase proteins [8,19,20]. Over time these changes can result in marked differences between isolates and lead to differences in host response to virus exposure and is often referred to as antigenic drift. Influenza is a segmented virus that allows for gene reassortment, where two viruses that infect the same host cell can create progeny viruses with gene segments from both parents. This dramatic shift in a virus is called antigenic shift, and although rare is thought to be a major contributor to human influenza pandemics. The ability to reassort gene segments provides for opportunities for novel viruses with unique phenotypic characteristics to emerge rapidly in the host [19].

Influenza viruses can have a wide host range and are believed to easily be able to jump to new host species, but rarely does the virus replicate and transmit well enough to sustain an infection in the new host [19,21]. Part of the low barrier to jumping to a new host is that influenza viruses use their surface hemagglutinin protein to attach to sialic acid as a host receptor. Sialic acid is the terminal sugar on N- and O- linked glycoproteins which are present on most host proteins in birds and mammals allowing for a ubiquitous host cell receptor. There are many types of sialic acid and the relative binding affinity of the viral hemagglutinin proteins changes depending on the type of sialic acid, which provides for one factor that selects for changes in host specificity [8,19]. For most AIVs they will bind with higher affinity to the α2,3-linked sialic acid receptors found in the respiratory and gastrointestinal tract of birds [8,19,22]. Most humans have α2,6-linked sialic acid in their upper respiratory tract and human adapted influenza viruses will bind with higher affinity. Many H9N2 G1 and Y280 lineage poultry isolates currently circulating have mutations that result in a high affinity for the α2,6-linked sialic acid receptor [19,23,24,25]. This change in receptor affinity of G1 and Y280 lineage viruses increases the potential that they may infect humans or other mammals without any additional change in receptor binding affinity [23]. The ability of H9N2s to infect humans without changes in receptor binding affinity increases the likelihood of transmission from birds to humans providing opportunity for genetic reassortment between avian and human influenza viruses. Additionally, a study on the type and prevalence of different conformations of the sialic acid receptors found that some poultry species have both α2,6-linked and α2,3-linked sialic acid receptors in the respiratory tract [19]. These poultry species including quail, turkey, and pheasants which are found in live bird markets (LBMS) and could also be hosts for reassortment and transmission to other species including humans [12]. This is of particular interest as monitoring of LBMs shows a high detection rate of multiple AIV subtypes and the close contact between humans and poultry provide ample opportunities for zoonotic transmission of AIVs [26].

An analysis was made of the variation of amino acids at position 226 (H3 numbering) of the hemagglutinin protein because of the known importance of this position on hemagglutinin binding to sialic acid. Through numerous studies, it has been shown that amino acids at positions 226 and 228 (H3 hemagglutinin numbering) are critical for determining the affinity for α2,3 or α2,6 sialic acid binding with glutamine (Q) for avian adapted viruses and leucine (L) for human adapted viruses at position 226. Analysis of H9N2 sequences available in GenBank was performed to identify amino acid variations at positions 226 and 228 using the Influenza Research Database [27]. At position 228 there was almost no variation with glycine being found in avian, human, and swine H9N2 viruses. Glycine is considered the amino acid associated with α2,3 sialic acid (avian binding). However considerable variation was seen with amino acids at position 226, but with rare exceptions only glutamine or leucine were found at this position. For all the H9N2 viruses from North America and the Korean lineage, only glutamine was observed. For simplicity of analysis, the H9N2 viruses were divided into viruses from China or from the rest of the world, not including North America or Korea. Although not a perfect correlation, this separates the viruses based on either the Y280 (China) or the G1 lineages (rest of world). Because of the availability of a high number of Y280 and G1 poultry sequences after the year 2000, these viral sequences were separated into three year increments and the percentage of viruses with glutamine or leucine were compared. The human viruses, with two exceptions, had the human adapted change of leucine. Swine, which are reported to have both α2,3 and α2,6 receptors on their respiratory epithelium, had an almost equal split of glutamine and leucine at position 226. For the Y280 and G1 lineage avian viruses, a major shift occurred with a majority of poultry viruses having glutamine before 2000, but after 2000 a shift occurred where most viruses in the G1 and Y280 lineage had leucine at this position (Table 1).

The presence of leucine at position 226 is thought to be a risk factor for viruses to be able to jump from birds to humans. It is unclear why there was a shift to leucine at this position in the poultry viruses, but similar changes have been seen with Chinese H7N9 viruses with a high percentage of avian viruses having leucine at the same position and an even higher percentage of human H7N9 viruses having leucine at the position [28]. There appears to be a selective pressure for viruses infecting humans to have leucine at this position. For the avian viruses, most wild bird viruses have glutamine at this position and the Korean lineage has maintained glutamine at this position for over 16 years. There does not appear to be a selective advantage for the viruses in the Y280 or the G1 lineage to maintain the avian-like glutamine at position 226 as more than 90% of recent viruses have leucine at this position. Table 1 clearly shows the shift over time from glutamine to leucine in these lineages of viruses, and these are the lineages that have infected humans.

Vaccination has been implemented in many countries where H9N2 is endemic in poultry. Vaccine pressure is another potential source of antigenic drift in poultry adapted H9N2 viruses as neutralizing antibody will provide strong selection pressure for variant viruses to emerge. Vaccination for H9N2 in Korea decreased the genetic diversity of H9N2 AIVs by virtually eliminating one of two circulating sublineages (clade A) from circulation, but the cocirculating clade B viruses, which were antigenically divergent from the vaccine, increased in prevalence, increased its antigenic diversity, and become the dominant circulating strain [29].

Many novel AIVs have been created through genetic reassortment as a result of the segmented nature of AIVs. Genetic reassortment provides opportunities for major changes in the phenotype of a virus, including the ability to infect and transmit in a new host. When the reassortment includes the hemagglutinin gene of different subtypes, it creates the possibility of a new virus that is immunologically new to the host population creating a potential pandemic situation.

The reassortment of gene segments has been frequently observed with H9N2 and other endemic influenza viruses. Mapping of the phylogenetic relationships of H9N2 isolates showed three major genotypes in chickens that have different gene constellations: genotype A was prevalent in the 1990s, but was replaced by H which was presumed to be better adapted to poultry and was the dominant genotype until the mid-2000s. Genotype H was then replaced by a dominant genotype that includes G1-like PB2 and M genes with an F/98-like backbone [14]. The S genotype shows greater infectivity, has caused more economic losses in poultry, and are the H9N2 donor viruses for the internal genes donated to H5 and H7 HPAIVs [14,30,31]. The H7N9 outbreak in China that started in 2013 had all six internal genes that originated from H9N2 viruses, and this new lineage of virus infected both poultry and humans [28]. Additional examples of reassortment viruses in humans have included H5N1, H5N6, and H10N8 viruses [32,33]. The presence of these highly adapted internal viral genes appear to again increase the possibility for new viral subtypes to emerge that can potentially infect poultry and people. Other changes with the current dominant genotype (S) include increased titers, shedding time, isolation rate, and preference for the α2,6-linked sialic acid receptor prevalent in humans [14,34]. The H9N2 poultry adapted lineages have also served as a donor of viral genes that have created unique viruses with poultry and zoonotic implications.

## 4. H9N2 in Mammals

The H9N2 viruses have been detected in a variety of mammalian species including humans, swine, dogs, weasels, and mink. The detection of virus from many mammalian species have been as part of routine surveillance of healthy animals, but in several cases were associated with clinical disease. Because of the concern about zoonotic infection with H9N2 viruses, many different animal models have been used to evaluate their potential for infection for humans. Unfortunately no single animal model species has proven to be a reliable predictor of human infections, but these studies do highlight the risk of human infection.

Swine have been a major concern for spread of influenza to humans because they are potential “mixing vessels” for avian, swine and human influenzas because they contain both α2,3 and α2,6 sialic acid in their upper respiratory tract and can potentially be infected by both avian, human, and swine influenza viruses. Novel viruses could emerge in swine through drift or shift potentially leading to pandemic strains [14,35]. In the late 1990s, H9N2 was isolated in swine and during the first decade of the 21st century, it was common to find H9N2 in swine in many parts of China [14]. As many as ten genotypes of H9N2 have been characterized in swine in China [35]. Swine experimentally infected with A/Chicken/Hong Kong/G9/1997 shed virus, but showed no signs of disease and did not transmit through direct contact [36]. Nearly 16% of pig serum samples collected in Guangdong Province and the Guangxi Zhuang Autonomous Region from 2010–2012 had antibodies for H9N2 with most of the positive samples reacting with Y280 lineage (97.4% of positive samples) virus antigen and only few reacting with G1 lineage (3.6% of positive samples) viral antigen by hemagglutination inhibition (HI) assay with [37]. A smaller portion of these samples were positive for anti-H9 neutralizing antibodies in virus neutralization tests, but the HI results suggest infection with avian H9N2 in swine [37]. Pigs inoculated with Y280-like isolates A/Chicken/Hong Kong/SSP177W/2009 or A/Chicken/Hong Kong/YU341/2008 shed virus and all seroconverted [37]. While pigs inoculated with G1-like A/Chicken/Hong Kong/NT10/2011 or A/Chicken/Hong Kong/NT449/2007 all shed, but only two pigs in the 2011 group seroconverted [38]. Additionally, higher titers were seen in the pigs inoculated with Y280-like isolates [37]. Transmission to naïve direct contact and indirect contact pigs did not happen in pigs inoculated with A/Chicken/Hong Kong/NT449/2007 (G1-like) or A/Chicken/Hong Kong/SSP177W/2009 (Y280-like) [37].

Mice infected with A/Guinea Fowl/Hong Kong/WF10/1999 (Y280-like), A/Guinea Fowl/Hong Kong/NT184/2003 (G1-like), or A/Quail/Hong Kong/G1/1997 resulted in systemic replication and a lethal infection, while A/Duck/Hong Kong/Y439/1997 only replicated in the respiratory system [23,36]. Using in vitro and in vivo methods, changes that alter host specificity have been found in the amino acid sequences of H9N2 isolates. In one study, mice challenged with a H9N2 duck isolate became infected and after a few passages in mice the virus became more virulent [39]. Multiple amino acid substitutions were acquired after many passages in mice resulting in a mouse-adapted virus that was highly virulent [39]. Two amino acid changes in the HA gene of A/chicken/Saudi Arabia/CP7/1998 were found after three passages in swine differentiated epithelial cells [40]. Using a related H9N2 isolate A/chicken/Emirates/R66/2002 (R66), three recombinant R66 isolates were made containing one, or both HA mutations to determine the importance of these HA mutations on sialic acid binding specificity and viral replication [40]. The A190V mutation reduced the specificity of the HA protein, changing the recognition from primarily α2,3-linked sialic acid to include α2,6 and α2,8/9-linked sialic acid and enhanced viral replication in cell culture [40]. The double mutation, A190Vand T212I increased the pathogenicity of the virus in mice, but did not affect the ciliary activity in swine epithelial cells in the manner expected of a strain virulent in swine [40].

Outbreaks of H9N2 in farmed mink in 2013 associated with clinical respiratory disease has been reported [41]. The isolates when used for experimental studies in mink showed that mink could be infected, shed virus, but exhibited only mild disease. Additional viruses were isolated from mink in 2015 with respiratory disease, and in experimental infections in mink the virus was able to infect mink and transmit to contact controls with some evidence of clinical disease. The farm where the virus was isolated also farmed raccoon dogs and foxes and some of these animals had seroconverted by HI to H9N2, and experimental inoculation showed seroconversion in these species but with no detectable shedding [42]. In a serosurvey of farmed mink in Shangdong province of China in 2013 from 5 different farms, both young and adult mink had around a 50% positive rate by HI. Experimental studies using a 2008 chicken H9N2 virus demonstrated virus infection, shedding and some pathogenicity with this virus isolate [43]. It is common practice to feed farmed mink raw whole chicken, which provides ample opportunity for exposure of mink. So the available data strongly supports the susceptibility of mink to H9N2 infection, but it remains unclear whether infections are occurring because of exposure to infected poultry or a mink-adapted H9N2 has emerged that is circulating in China.

Ferrets have become the preferred mammalian model for influenza viruses because they can be infected and show clinical respiratory disease with both human seasonal and novel influenza viruses. Ferrets are also widely available, so multiple studies have been performed with H9N2 viruses in ferrets. Ferrets challenged with a H9N2 isolates from multiple lineages (North American, G1, and Y280) and hosts (human, mammalian, and avian) exhibited viral shedding at similar levels [7]. Transmission to direct contacts occurred in four groups and no group transmitted by aerosol: A/Human/Hong Kong/33982/2009 and A/Chicken/Hong Kong/G9/1997 were transmitted to both direct contacts, A/Human/Hong Kong/1073/1999 and A/Swine/Hong Kong/9A-1/1998 transmitted to one direct contact [7]. Transmission to contacts by A/Chicken/Hong Kong/G9/1997 was delayed, shedding was not detected until five days post-contact (dpc), and virus was shed at lower titers compared to the human origin isolates who displayed viral shedding by 3 dpc [7]. While the human origin isolates had greater virulence and transmissibility than the other H9N2 isolates, the results suggest that internal genes may be important for viral replication in human and mammalian models. Having leucine or glutamine at HA-226 did not change virus transmissibility and none of the isolates in this study had the PB2 E627K substitution [7]. The E627K substitution of lysine for glutamic acid on the PB2 gene is an important host factor that enhances viral replication of AIVs from infecting humans in the respiratory tract of mammals [44,45]. Specifically, the presence of lysine reduces the optimal temperature the polymerase requires for viral replication, allowing efficient replication of AIVs in the respiratory tract of humans at 33 °C and can result in increased pathogenicity of AIVs in humans and other mammals [44,45]. This increased polymerase activity is nearly five times higher in enzyme catalysis at 34 °C and decreased replication efficiency at higher temperatures [46]. Ferrets challenged with five wild type H9N2 isolates, collected from 1988–2003 showed some limited lethargy and body temperature elevation, but had no overt signs of disease and only two isolates infected contact ferrets [47]. Site-directed mutagenesis of the amino acid at position 226 in the HA receptor binding site altered replication and transmission of H9N2; isolates with leucine instead of glutamine at position 226 showed better replication of virus and transmission to contact ferrets [47]. Ferrets challenged with a reassortant of avian-human H9N2 had clinical signs of disease including sneezing, transmitted efficiently to direct contact ferrets, but not by aerosol contact [47]. Ferrets were used to examine the pathology of a novel H9N2 reassortant isolated from a human with influenza-like illness (ILI) and two environmental H9N2 isolates collected from LPMs [48]. Phylogenetic analysis of the HA and NA genes of these three isolates placed two, A/environment/Jiangxi/02898/2012 and A/hunanlengshuitan/11197/2013, in the Y280 HA and NA gene lineages, while A/environment/Sichuan/11/2010 was in the G1 lineage for the HA gene but was in the Eurasian lineage for the NA gene [48]. Both Y280-like isolates replicated well in ferrets, but the human isolate caused higher titers at 5 days post inoculation, two days earlier than the environmental isolate [48]. Additionally, although both isolates caused gross lesions in the lungs, viral shedding from nasal turbinates, trachea, and lung tissue samples was significantly higher in the group infected with the human isolate [48]. The G1-like environmental virus did not shed detectable virus from the upper respiratory tract but did show some positive results by qRT-PCR and the ferrets seroconverted, showing a fourfold increase in titer [48]. These serology titers were significantly less than those found in the groups challenged with the Y280-like isolates [48]. Finally, while nasal discharge was the only clinical sign exhibited by inoculated ferrets, A/environment/Jiangxi/02898/2012 and A/hunanlengshuitan/11197/2013 both induced mild bronchopneumonia [48].

Guinea pigs infected with A/Chicken/Shandong/Li-2/2010, A/Chicken/Shandong/Li-3/2010, or A/Chicken/Jilin/Hu-3/2006 shed virus but did not transmit to contacts [49]. After 15 serial passages with A/Chicken/Shandong/Li-2/2010, which recognized both α2,3 and α2,6-linked sialic acid receptors, an adapted isolate with five amino acid changes was able to transmit efficiently to all direct contacts, but could not transmit through aerosol to naïve contacts in an adjacent cage in guinea pigs or ferrets [49]. The combination of substitutions NP-E434K and HA1-Q227P or HA2-D46E were the key to transmission to contacts [49]. Additionally, having both HA substitutions increased binding affinity for both α2,3 to the α2,6-linked sialic acid receptors [49]. Amino acid substitutions NP-E434K and PB2-D195N were responsible for a 12.5 times increase in polymerase activity in P15 when compared to the parent isolate [49].

## 5. H9N2 in Humans

Seasonal influenza infections in humans are a regular occurrence as a result of influenza type A (H3N2 and H1N1), B, or C infections [50,51]. Specific barriers such as receptor binding affinity, the temperature range of viral polymerase, and contributions from other viral genes all have a role in preventing AIVs from infecting humans [52]. While AIVs occasionally transmit directly from birds to humans, these infections are sporadic [14,50,51] and usually do not transmit efficiently human to human. However, antigenic shifts in influenza viruses are dangerous as they can result in novel viruses for which humans have no immunity [50,51,53]. A novel virus with the ability to spread amongst humans could result in a pandemic [51,53]. Three of the four pandemic influenza outbreaks in the 20th century were the result of genetic reassortment events that included AIVs [53,54].

Although sporadic, the continuing occurrence of H9N2 infections in humans provides a chance that pandemic H9N2 strains may develop [8,55,56]. Humans in close contact with poultry are at an increased risk of AIV exposure and many recent H9N2 isolates have acquired adaptations that increase human susceptibility. Understanding how prevalent H9N2 infections are in humans and the origin of these infections will provide a better ability to prevent the spread of H9N2 to humans and ultimately prevent human-to-human transmission.

The first known human H9N2 infection was discovered in southern China in 1998 and additional cases have been reported in the years since [14,55,56,57,58]. Currently 24 H9N2 viruses isolated from humans have been sequenced (Table 2). Mild illness is characteristic of most H9N2 infections in humans with symptoms consisting of fever and coughing [55]. Because of widespread H9N2 infections in poultry, particularly in live bird markets, a large number of humans have likely been exposed by direct or indirect contact with poultry. Since the documented cases of human infection has generally only been mild respiratory disease, it is likely that many cases of H9N2 human infections have occurred and either were subclinical infections or were not severe enough to result in the infection being diagnosed by a physician.

### 5.1. Seroprevalence of H9N2 in Humans

Multiple studies (Table 3) have looked at the seroprevalence of H9N2 antibodies in humans and this evidence suggests human infections commonly occur in countries with endemically infected poultry [54,59,60,61,62,63,64,65,66,67,68,69,70,71,72,73,74,75,76,77,78,79,80,81,82,83,84,85,86,87,88,89]. For all human studies, the serology results are reported as titers from hemagglutination inhibition (HI) assays and/or microneutralization (MN) assays. A summary is presented (Table 3) using the calculated percent positive based on samples split between exposure categories: occupational exposure (people that work in the poultry or healthcare industry) vs general exposure (limited exposure to poultry or infected people). There were differences in antigens used for each study, and in the species of red blood cells used for the HI assays that could result in differences in sensitivity for the tests [90].

In the USA where H9N2 is not enzootic in poultry, there is an extremely low rate of serological positive samples for H9N2 infections in poultry workers or the general populations that reflects the lack of exposure to the virus [64,65]. Pakistan, Iran, and China where H9N2 is endemic in poultry had the highest percentage of seropositive samples of all the studies (Table 3). Occupational exposure samples were consistently more likely to be seropositive than the general exposure samples. In Pakistan, farm workers from different areas of poultry production showed nearly 50% seroprevalence of H9N2 antibodies with a conservative HI cutoff of 1/160 [84]. Workers with the highest risk of H9N2 infection in Pakistan were those on breeder farms where birds were floor-reared [84]. Samples from Southern China, where there is a greater concentration of live poultry, had significantly higher seroprevalence than those from Northern China [85]. While most clinical cases of H9N2 infection have been in children, adults and the elderly also had a high seroprevalence of H9N2 [85]. Poultry workers that had contact with live birds had a much higher prevalence of H9N2 infection than those that worked in slaughtering plants or in wild bird habitats [85]. In Iran, all studies showed occupationally exposed groups also had a higher seropositive rate than general population samples [62,67,69,79,86]. While one study showed only 1.6% positive by HI, samples were 11.5% positive by ELISA [67]. One study on hospital workers showed nearly 33% of samples were seropositive compared to 2.5% for the general public suggesting that contact with infected people could be the source [69]. However, this study did not include whether these subjects had any poultry exposure and since human-to-human transmission of H9N2 has not been shown to happen frequently this conclusion is not supported. Additionally, there was no mention of checking for cross-reactivity with H3N2 to show the results were only for H9N2 antibodies. Another study suggests that direct poultry contact results in more seropositive results against H9N2 than healthcare workers and control subjects: poultry workers had nearly double the amount of positive serology as healthcare workers and the general population [79]. The most recent Iran study used two antigens: one representative of G1 sublineage Clade A (equal split Leu226 and Gln226) and one representative of G1 sublineage Clade B (more than 90% of isolates Leu226) which is currently circulating in the country [62]. Only Clade B showed seropositive results in the generally exposed group and had 12% positive versus 2% in the occupationally exposed group [62]. 

In Vietnam where H5 and H9 isolates are endemic in poultry, there is a low human seroprevalence [59]. Serological examination of healthy individuals with exposure to poultry at three different time points detected H9 positive samples under 10% of the time [59]. Seroprevalence of H9N2 antibodies in Indian poultry workers not vaccinated against seasonal or pandemic influenzas found nearly 6% were positive [70]. A serological survey of Romanian adults with exposure to poultry, wild birds, or swine found that around 9% were seropositive for H9N2 antibodies [89]. These results were not a result of antibodies to pandemic H2N2 or human H3N2 and was notably related with household or community poultry exposure [89]. While surveillance of AIVs in poultry is nonexistent in Romania, the prevalence of H9N2 antibodies in humans exposed to poultry suggests it may be present [89]. Serological findings from many different countries suggest that H9N2 infections in humans are more common than what is reported and poultry exposure in endemic countries, is an important risk factor in H9N2 infections [50,55,56,57,58,59,60,61,62,63,64,65,66,67,68,69,70,71,72,73,74,75,76,77,78,79,80,81,82,83,84,85].

### 5.2. Molecular Characterization of Human H9N2 Isolates

Currently there are 24 human H9N2 influenza virus sequences available in GenBank or GISAID that allows for some genetic comparisons (Table 2). As previously identified in Table 1, most of the human viruses have leucine at position 226 of the hemagglutinin protein that is correlated to human adapted influenza viruses. In addition other markers of human adaptation have been identified in the PB2 and PB1 polymerase genes. The marker at position 627 of the PB2 is one of the best characterized where most avian strains have glutamic acid at this position and human adapted viruses have lysine at this position. One selective advantage that lysine has at this position is increased polymerase activity at a lower temperature. Because birds have a higher body temperature, the glutamic acid at position 627 works better at the higher temperature. Human’s virus replication is mostly in the upper respiratory tract where the temperature is lower and the lysine mutation allows more efficient polymerase activity. In an analysis of avian H9N2 viruses and human H9N2 isolates, 4 amino acids, all in the PB2 protein, were identified where the human isolates had a higher percentage of human adaptive changes than the avian viruses (Table 4). All of the human adaptive mutations were found in the avian strains at least at a low level. However, over 11% of the avian isolates had valine at position 588 that is considered the human adaptive amino acid. This change is also associated with polymerase activity [91]. Although the percentage of most of these human adapted markers appear to be low in poultry populations, the presence of these changes appears to increase the likelihood of viruses that can more easily transmit to humans. Because the H9N2 viruses are so common in poultry, the increased ability for viruses to infect humans and the wide presence of viruses appears to be a serious threat for viruses that may eventually transmit efficiently in humans.

**Figure 1:**
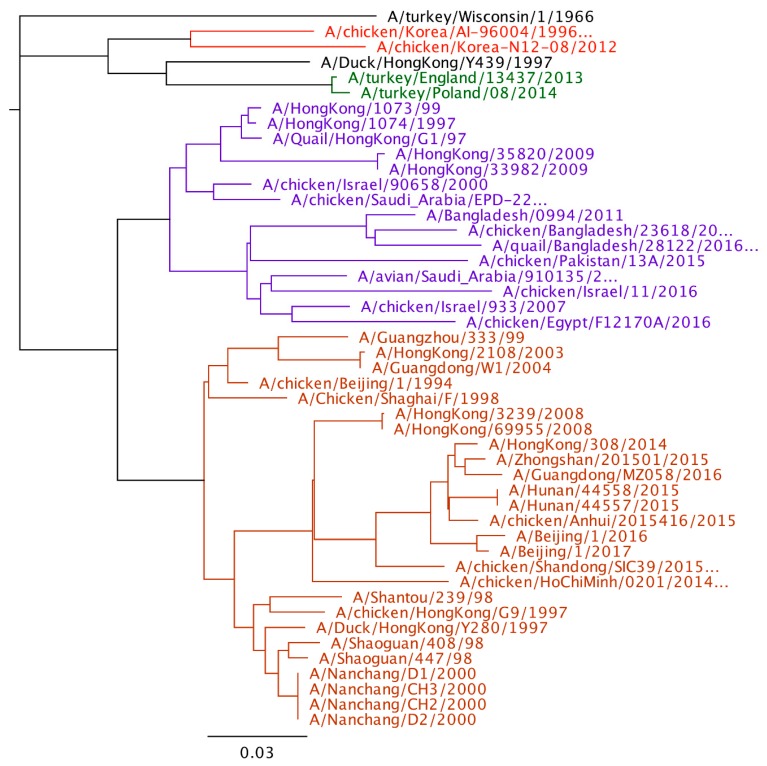
Phylogenetic tree of H9 hemagglutinin gene (Geneious 11.1.5). The four major poultry adapted lineages are color coded. Red-Korean lineage, Green-European lineage, Purple-G1, and Orange-Y280.

Phylogenetic analysis of H9N2 viruses show large genetic diversity and at least 4 poultry adapted lineages of virus as mentioned earlier. When comparing the human viruses to the poultry viral lineages, all the sequenced viruses from human are in the Y280 or the G1 lineage. No human cases have been reported from the Korean, European, or wild bird lineages. Although more viruses have been reported from the Y280 lineage, it is unclear whether this represents a greater zoonotic risk of the Y280 lineage compared to the G1 lineage or there is just an increased likelihood of detection in Y280 virus infected humans compared to humans in G1 infected countries. The last G1 virus from humans was in 2011, but Y280 lineage viruses have been reported as recently as 2017 (Figure 1).

## 6. H9N2 Prevention

The best way to prevent human infections are to prevent exposure to the virus. Because H9N2 viruses are endemic in so many countries, it is unlikely that control measures such as test and slaughter approaches can be used because of the high economic cost. Reducing the exposure to humans is possible with the understanding of where the exposure is occurring. In countries where live bird markets play an important role of marketing of poultry to the consumers, most people will only be exposed to poultry and H9N2 virus while they are shopping or visiting close to the LBMs. Efforts to reduce infected poultry in the marketing system therefore should be a priority.

The use of vaccination of poultry likely provides the most practical control tool to reduce human exposure. Traditional vaccines for AIVs are made from influenza isolates grown in embryonated chicken eggs (ECE), inactivated, and delivered with mineral oil adjuvant. The use of high quality, antigenically matched and properly applied vaccines can greatly reduce clinical disease in poultry and of equal importance can greatly reduce or eliminate virus shedding in birds that do get exposed to the virus. This reduction of virus being shed into the environment will greatly reduce the human exposure. While antigenically matched vaccines can provide good protection against clinical signs of disease and reduce viral shedding, this protection does not extend to less genetically related isolates of the same subtype and does not prevent infection of other AIV subtypes [92]. Many countries where H9N2 is endemic have been using vaccination as a control method, but many still see outbreaks resulting from H9N2 AIVs [93]. New vaccine developments are working on reducing the cost and difficulty of production of current inactivated vaccines while also increasing the efficacy and providing broader protection against multiple H9N2 subtypes. Recombinant viral vectors and virus like particles (VLPs) are new options being explored in vaccine development. Production of VLPs is achieved by cell culture, does not require ECEs to produce vaccines and can be used to produce immunogenic vaccines for multiple AIV subtypes [94,95]. Intranasal immunization of ferrets with VLPs containing H5, H7, and H9 proteins significantly reduced viral load after challenge with three AIVs including an H9N2 human origin isolate (human/HK/33984/2009 [94]. A recombinant Newcastle Disease virus (NDV) H9 vaccine that expressed multiple H9N2 HA was successful in protecting against heterologous H9N2 viruses in chickens [93]. Reduction of viral shedding and elimination of clinical signs post challenge was achieved although genetic differences between the vaccine HA and the challenge strain were still impactful with less protection from a more distantly related isolate [93]. Further research is needed to increase the availability of these new vaccines for use in poultry. There have been two recent examples where vaccination has reduced the number of human infections. The first was in Vietnam in 2004 where H5N1 HPAI virus was causing severe disease in the poultry industry and a high number of human infections were occurring. Vaccination was used in poultry in a government sponsored vaccination program, and the incidence of human infections greatly decreased, although the H5N1 virus remained endemic in the country. A similar government sponsored vaccination program in China in 2017 against H7N9 AIV also appears to have greatly decreased the number of human infections.

Other practical tools should be considered to reduce human exposure in live bird markets. At least with H5N1 HPAIV, it has been shown that the slaughter and defeathering procedures aerosolizes a large amount of virus. Efforts to control this release of virus with tools as simple as the use of plastic bags to contain birds during the slaughter process can potentially be helpful to reduce environmental exposure [96]. Use of centralized slaughtering facilities with maintenance of a cold chain to deliver fresh meat to the consumer will also greatly reduce the opportunity for human exposure. In a recent comparison in China, 39.8% of chicken carcasses from live bird markets had detectable avian influenza virus while none of the carcasses supplied to supermarkets were positive [97].

Vaccination for humans is currently not a practical option for H9N2 avian influenza because the clinical disease currently observed doesn’t justify the expense. In addition because of the wide antigenic variation observed between and within lineages, a single vaccine would likely not protect for all potential H9N2 exposures. However, H9N2 viruses are considered as a legitimate pandemic risk and some preparation has been conducted by the World Health Organization Global Influenza Surveillance and Response System to prepare Candidate Vaccine Viruses (CVV) that can be used to speed the process of vaccine production if a pandemic begins to emerge. These CVVs include six either wild type viruses or reverse genetics produced viruses that are antigenic, grow to reasonable titer, and are free of known adventitious pathogens. In addition reagents for potency testing are also available [98]. The availability of these seed strains, if properly antigenically matched to the pandemic strain, can potentially save valuable time in the preparation of vaccine in an emerging pandemic.

## 7. Conclusions

As one of the most prevalent AIV subtypes circulating in poultry around the world, H9N2 should be at the forefront of efforts to prevent its spread amongst poultry and the secondary spread to humans and other species. The Y280 and G1 lineages of H9N2 are poultry adapted and have spread throughout Eurasia and Africa and have become endemic in many countries. Preventing a pandemic with H9N2 is possible with the right strategy for controlling H9N2 in poultry. This includes implementation of monitoring programs, vaccination, good biosecurity at farms and LBMs as well as implementing rest days at LBMs. All these efforts to reduce the number of H9N2 infections in poultry and prevent the spread of the virus to mammals and humans will require a lot of resources. If we can control the transmission of H9N2 in poultry and prevent humans from contracting this disease, we will greatly reduce the risk of human-to-human transfer and potentially prevent a pandemic.

## Figures and Tables

**Table 1:** Comparison of amino acid variation at position 226 (H3 numbering) of H9N2 isolates from different geographic regions.

Origin	Glutamine	Leucine	Total Isolates
North America 1966–2017 ^a^	100% ^b^	0%	68
South Korea 1996–2012	100%	0%	128
China 1980–1997	59.50%	40.50%	42
China 1998–2000	39.80%	60.10%	153
China 2001–2003	25.70%	74.30%	268
China 2004–2006	26.70%	73.30%	236
China 2007–2009	14.20%	85.80%	513
China 2010–2012	6.90%	93.10%	1182
China 2013–2015	2.80%	97.20%	688
China 2016-2018	3.00%	97.00%	66
rest of world 1980–2000	61.10%	38.90%	90
rest of world 2001–2003	32%	68%	147
rest of world 2004–2006	32%	68%	128
rest of world 2007–2009	30.70%	69.30%	150
rest of world 2010–2012	9.50%	90.50%	190
rest of world 2013–2015	4.20%	95.80%	237
rest of world 2016–2018	19.10%	80.90%	157
swine 1998–2015	54.2%	45.8%	48
human 1998–2016	8.7%	91.3%	23

^a^ All viruses are avian unless otherwise indicated. ^b^ The comparison was made only for isolates with glutamine or leucine at position 226 (H3 numbering). A small number of isolates, which had other amino acids at position 226, including methionine, were excluded from the percentage calculations.

**Table 2:** Human H9N2 isolates.

H9N2 Sequenced Human Viruses	Poultry Lineage
A/Shaoguan/447/1998	Y280
A/Shaoguan/408/1998	Y280
A/Shantou/239/1998	Y280
A/Hong Kong/1073/1999	G1
A/Hong Kong/1074/1999	G1
A/Guangzhou/333/1999	Y280
A/Nanchang/CH3/2000	Y280
A/Nanchang/D1/2000	Y280
A/Nanchang/D2/2000	Y280
A/Nanchang/CH2/2000	Y280
A/HK/2108/2003	Y280
A/Guangdong/W1/2004	Y280
A/Hong Kong/3239/2008	Y280
A/Hong Kong/69955/2008	Y280
A/Hong Kong/35820/2009	G1
A/Hong Kong/33982/2009	G1
A/Bangladesh/0994/2011	G1
A/Hong Kong/308/2014	Y280
A/Hunan/44557/2015	Y280
A/Zhongshan/201501/2015	Y280
A/Hunan/44558/2015	Y280
A/Beijing/1/2016	Y280
A/Guangdong/MZ058/2016	Y280
A/Beijing/1/2017	Y280

**Table 3:** H9N2 human serology studies.

Country	Dates Sampled	Occupational Exposure	Percent Positiv (%)	General Exposure	Percent Positive (%)	HI Cutoff	Occupational Exposure	Percent Positive (%)	General Exposure	Percent Positive (%)	MN Cutoff	Ref.
China ^1^	1999						0/54	0	1/110	0.91	1600	[87]
Vietnam ^1^	2001						1/200	0.5	0/200	0	1/40	[72]
UnitedStates ^2^	2004						1/385	0.26	1/484	0.21	1/40	[64]
China ^1^	Apr. 2006–Feb. 2008	12/1060	1.1	0/407	0	1/160						[80]
Iran ^1^	Nov. 2006	48/127	37.7	0/25	0	1/20						[86]
UnitedStates ^2^	Mar. 2007–Apr. 2008						4/93	4.3	1/78	1.28	1/10	[82]
China ^1^	Mar. 2007–Jul. 2008	95/1890	5.03	4/301	1.33	1/20						[73]
China ^1^	Jan. 2008–Dec. 2010	103/840	12.47	47/1663	2.83	1/40					1/20	[54]
Cambodia ^3^	Apr. 2008–Oct. 2008								21/777	2.7	1/10	[60]
Thailand ^3^	Apr. 2008–Oct. 2010 Enrollment								38/800	4.7	1/10	[88]
	12 Month								21/768	2.7	1/10	[68]
	24 Month								40/784	5.1	1/10	
Nigeria ^2^	Dec. 2008–Jun. 2009						4/316	1.27	0/54	0	1/10	[61]
United States ^2^	2009–2010						1/157	0.63	0/78	0		[65]
China ^1^	2009–2011	1912/14,896	12.8			1/40	453/14,896	3.04			1/40	[85]
		489/13,453	3.6				159/13,453	1.18				
Romania ^2^	Feb. 2009–Jan. 2010	31/312	0.7	2/51	3.92	1/40	29/312	10.14	4/51	5.19	1/10	[89]
China ^1^	Mar. 2009–Dec. 2012	37/2006	1.8	0/83	0	1/160	24/37	64.86	0			[74]
China ^1^	May 2010			18/1039	1.73	1/40						[75]
India ^1^	Jul. 2010–Dec. 2010	21/338	6.2	0/249	0	1/40	19/338	5.6			1/40	[70]
Iran ^1^	Dec. 2010–Jul. 2011	3/182	1.6	0	0	1/20						[67]
Pakistan ^1^	2010–2011	165/384	43.0			1/160						[84]
China ^1^	Jan. 2011–Dec. 2013	51/600	8.5	11/600	1.8	1/40	51/600	8.5	11/600	1.8	1/40	[78]
China ^1^	Mar. 2011	12/1741	0.7			1/40						[63]
China ^1^	Dec. 2011–Feb. 2012	9/382	2.3	0/100	0	1/40	7/382	1.80	0/100	0	1/40	[76]
Iran ^1^	Sep. 2012–Jan. 2013	12/100	12.0	2/100	2	1/40	17/100	17	3/100	3	1/40	[62]
China ^1^	Oct. 2013–Jul. 2014	56/171	32.7			1/40						[81]
China ^1^	Dec. 2013–Jan. 2014	50/546	9.2	5/264	1.89	1/40						[77]
Vietnam ^1^	2013–2015	28/784	3.6	0	0	1/40						[59]
Iran ^1^	not reported	98/300	32.6	8/300	2.5	1/40						[69]
Iran ^1^	not reported	163/240	67.9	14/60	23.3	1/20						[79]
Pakistan ^1^	not reported	209/465	45.0	0/25	0	1/160						[83]
Egypt ^1^	not reported	0/60	0.0			1/40						[66]

^1^ H9N2 is endemic in poultry in this country. ^2^ H9N2 is not endemic in poultry in this country. ^3^ H9N2 endemic status is unknown in this country.

**Table 4:** Comparison of molecular markers of human adapted influenza strains.

Gene Segment	Avian > Mammalian	Avian Isolates	Human Isolates	Activity
PB2	K526R	3.20%	6.30%	increase polymerase activity, enhance virus replication
PB2	A588V	11.90%	25%	higher polymerase activity, increased virulence mice
PB2	E627K	0.80%	6.30%	associated with mammalian adaptation
PB2	D701N	0.05%	6.30%	aerosol transmission guinea pigs
